# Diffuse Alveolar Hemorrhage Associated with Edoxaban Therapy

**DOI:** 10.1155/2016/7938062

**Published:** 2016-10-31

**Authors:** Kenichi Nitta, Hiroshi Imamura, Akihiro Yashio, Satoko Kashima, Katsunori Mochizuki

**Affiliations:** Department of Emergency and Critical Care Medicine, Shinshu University School of Medicine, Nagano, Japan

## Abstract

*Introduction.* The main adverse effect of anticoagulant therapy is bleeding, and major bleeding, including intracranial, gastrointestinal, and retroperitoneal bleeding, has been reported as an adverse effect of edoxaban, a direct oral anticoagulant (DOAC). Bleeding during systemic anticoagulation with edoxaban presents a therapeutic conundrum, because there is currently no safe or efficacious reversal agent to stop major bleeding.* Case Report.* A 51-year-old woman had multiple traumatic injuries, including lower limb fractures. On day 8, she developed deep venous thrombosis, and edoxaban was administered orally. On day 38, she developed fungemia, which was treated with an antifungal drug. On day 43, she presented with dyspnea. Chest computed tomography scan showed bilateral diffuse ground-glass opacities in the whole lung fields. The results of the subsequent workup (i.e., serum levels of the antineutrophil cytoplasmic antibody, antinuclear antibody, and antiglomerular basement membrane antibody) and microbiological study were unremarkable. Based on these findings, her condition was diagnosed as diffuse alveolar hemorrhage (DAH) associated with edoxaban therapy. The lung opacities disappeared spontaneously after edoxaban therapy was discontinued.* Conclusion.* DAH is a dangerous complication associated with edoxaban therapy. DOACs, including edoxaban, should be prescribed with caution, especially for patients in a critical condition.

## 1. Introduction

Venous thromboembolism is the third most common cardiovascular disorder after coronary heart disease and stroke [1]. Treatment of the venous and arterial thrombotic phenomena is a main medical challenge, and the development of anticoagulant drugs is a revolution in medicine. Edoxaban, a direct oral anticoagulant (DOAC), was approved by the United States Food and Drug Administration in January 2015 to prevent stroke in patients with nonvalvular atrial fibrillation and treat deep venous thrombosis (DVT) and pulmonary embolism after 5 to 10 days of parenteral anticoagulation. The main adverse effect of anticoagulant therapy is bleeding, and major bleeding, including intracranial, gastrointestinal, and retroperitoneal bleeding, has been reported to be an adverse effect of edoxaban [2]. Bleeding during systemic anticoagulation with edoxaban presents a therapeutic conundrum, because there is currently no safe or efficacious reversal agent to stop major bleeding [3].

Diffuse alveolar hemorrhage (DAH) has not been previously reported to be a complication of edoxaban therapy. Herein, we describe a case of DAH associated with edoxaban therapy in a woman who suffered multiple traumatic injuries accompanied by DVT and a fungal infection.

## 2. Case Presentation

A 51-year-old woman with no relevant medical history was admitted to the emergency department with shock due to multiple traumatic injuries caused by a traffic crash. An occlusion balloon catheter was introduced temporarily from the femoral artery to the descending aorta. The patient simultaneously received blood transfusion, and the patient's cardiorespiratory condition gradually stabilized. Whole-body computed tomography (CT) scan showed injuries to the wall of the small and large intestines. Radiographic imaging demonstrated a right femoral shaft fracture and a left open tibial fracture. Consequently, the patient underwent resection of the small and large intestines, two colostomies, and osteosynthesis, after which the patient was admitted to the intensive care unit.

On the fourth day after admission (postoperative day 2), anticoagulant therapy with heparin was initiated to prevent DVT. On the eighth day after admission, the patient's D-dimer level increased, and contrast-enhanced CT scan showed DVT in the right femoral vein. Therefore, edoxaban (30 mg/day) was administered orally. Ordinarily, 60 mg edoxaban is administered once daily, but since the patient weighed 51 kg and had normal renal function, the dose was reduced.

**Figure 1:**
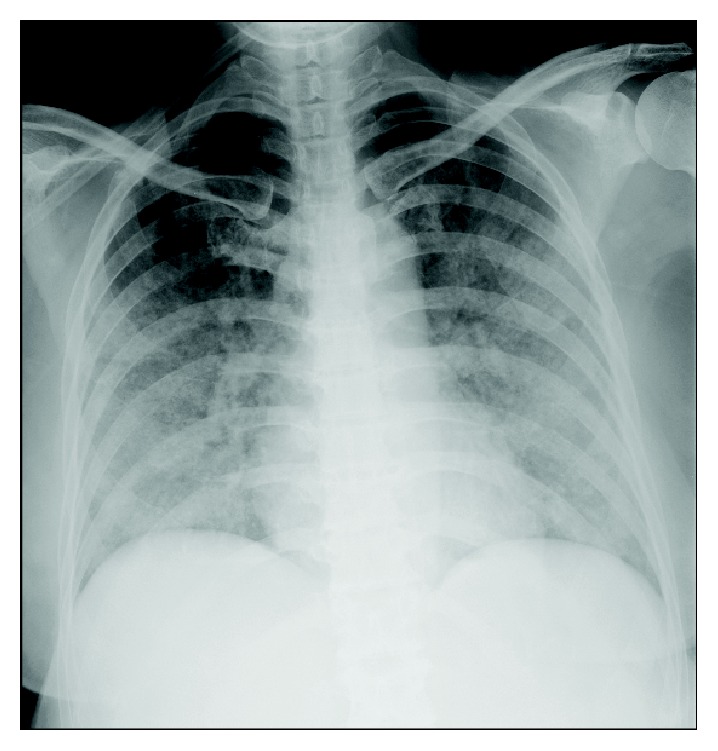
Chest radiograph showing infiltrates bilaterally.

**Figure 2:**
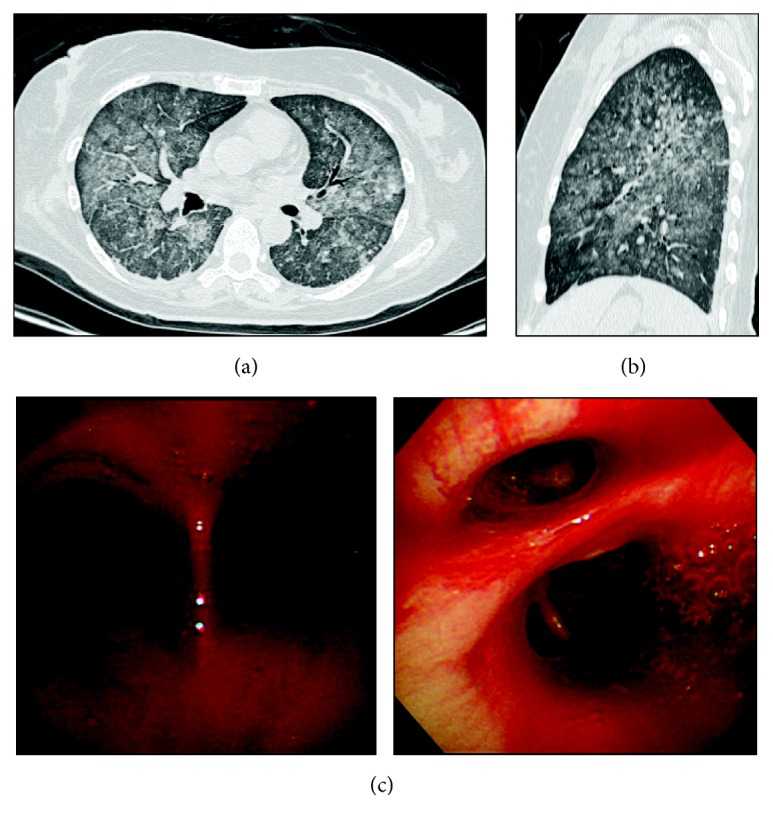
Imaging studies. Axial (a) and sagittal (b) chest computed tomography scans showing diffuse ground-glass opacities bilaterally. Bronchoscopy images (c) showing bloody discharge around the bifurcation of the trachea and the right intermediate and inferior bronchi.

On day 36, the patient developed a fever with nausea and vomiting. Blood cultures were performed, and a course of antibiotics was initiated for a possible intraperitoneal infection. On day 38, the patient's condition was diagnosed as sepsis due to* Candida guilliermondii*, and treatment with micafungin was initiated. On day 43, coagulation tests showed a prothrombin time (PT) of 16.2 s, prothrombin time-international normalized ratio (PT-INR) of 1.44, and activated partial thromboplastin time of 35.6 s. The patient had already received a dose of edoxaban (30 mg) that day. That night, the patient demonstrated dyspnea with tachypnea and had blood in her sputum along with an abrupt decrease in oxygen saturation. Chest radiography scan showed lung opacities bilaterally (Figure 1). The patient was intubated and underwent mechanical ventilation with positive end-expiratory pressure (PEEP) because of worsening hypoxemia. The arterial partial pressure of oxygen to fraction of inspired oxygen (PaO_2_/FiO_2_) ratio was 113 mmHg on PEEP with 6 cm H_2_O. Chest CT scans showed diffuse ground-glass opacities bilaterally (Figures 2(a) and 2(b)). At this point, the PT was 18.7 s and PT-INR was 1.66. Echocardiography results showed normal left ventricular function and no valvular disease. Serum levels of the antineutrophil cytoplasmic antibody, perinuclear antineutrophil cytoplasmic antibody, antinuclear antibody, anti-dsDNA antibody, and antiglomerular basement membrane antibody were negative. Bronchoscopy results showed bloody discharge around the bifurcation of the trachea and no malignant tumor (Figure 2(c)), and subsequent bronchoalveolar lavage (BAL) findings showed a bloody gross appearance. All results of the sputum cultures and BAL fluid did not indicate any causative pathogen. The patient had a pure DAH confined to the lungs with no evidence of disseminated intravascular coagulation or hemorrhagic events occurring elsewhere in the body. In addition, the patient did not receive any concomitant therapy with specific P-glycoprotein inhibitors (i.e., verapamil; quinidine; or short-term use of azithromycin, clarithromycin, erythromycin, oral itraconazole, or oral ketoconazole), which may increase the serum concentration of edoxaban. Finally, we diagnosed this case as DAH associated with edoxaban therapy, which was triggered by fungemia.

Conservative therapy with PEEP was continued, and the lung opacities disappeared spontaneously following the discontinuation of edoxaban therapy. The bloody sputum gradually diminished, and the patient's respiratory status recovered over time.

The patient was extubated on day 50, and the patient showed gradual improvement without DVT recurrence. Additionally, the patient could walk using a crutch. On day 84, chest radiography scan showed almost normal findings, and the patient was transferred to another hospital for leg rehabilitation.

## 3. Discussion

To the best of our knowledge, this is the first reported case of DAH associated with edoxaban therapy. DAH is a clinical syndrome resulting from extensive bleeding in the acinar portion of the lung [4], and it is caused by diseases that damage the alveolar capillary barrier or by coagulation disorders. Generally, DAH may develop in several pathologic conditions, including vasculitis, autoimmune diseases, acute poststreptococcal glomerulonephritis, and any pneumorenal syndrome. However, the incidence of DAH in patients receiving anticoagulant therapy is low [5]. DAH secondary to warfarin therapy has been rarely reported [6, 7], but DAH is a serious adverse effect of dabigatran [8] and apixaban [9]. However, DAH associated with edoxaban has not been reported in the literature. In our patient, the BAL fluid became progressively more hemorrhagic, with no evidence of massive bleeding or endobronchial pathology. In addition, tests for specific antibodies were negative, and although the patient had a fungal infection, all sputum and BAL fluid cultures were negative for any causative pathogen. Moreover, the patient's lung opacities disappeared spontaneously after edoxaban therapy was discontinued. On the basis of these findings, the patient's condition was diagnosed as DAH associated with edoxaban therapy, which was triggered by fungemia.

Routine coagulation tests are not conducted to determine the anticoagulation status of patients receiving DOACs. However, edoxaban and other factor Xa inhibitors prolong the clotting time tests, including the PT, PT-INR, and activated partial thromboplastin time (aPTT). Additionally, these clotting time tests are not indicative of bleeding risks, because there is no dose-response relationship that correlates the changes between PT and aPTT to a specific dose of DOACs [10]. As traditional coagulation assays do not reliably measure the anticoagulant effects of DOACs, viscoelastic assays may be a useful tool for assessing coagulation function in the presence of DOACs [11]. In the present case, although we monitored the patient's PT-INR daily after fungemia was diagnosed, we could not prevent DAH. One of the disadvantages of DOACs is physicians' limited experience with these drugs in cases of abnormal coagulation. Accordingly, DOACs should be used cautiously in patients who require invasive procedures [12] and intensive care.

Warfarin anticoagulation can be reversed. A recent study suggested that andexanet alfa can reverse the anticoagulant activity of apixaban and rivaroxaban in older healthy participants without clinical toxic effects [3]. However, there is still no safe or efficacious reversal agent to stop factor Xa inhibitor-associated acute major bleeding. The peak plasma concentration of edoxaban occurs 1 to 2 h after oral administration, and the usual plasma half-life is 8 to 10 h [13]. As DOACs have short elimination half-lives, time is the most important antidote. In many cases, a bleeding event can be managed effectively by simply providing supportive therapy and withholding the DOAC in question at least temporarily [14]. Fatal bleeding associated with edoxaban is particularly challenging, because there is limited experience with the expected clinical course. Fortunately, our patient's respiratory status improved spontaneously with the discontinuation of edoxaban therapy. Moreover, recent data from human studies have indicated that prothrombin complex concentrate (PCC) products may be viable agents for correcting coagulation changes associated with dabigatran, rivaroxaban, and apixaban [15, 16]. However, the use of PCCs may increase the risk for thrombosis; therefore, it is important to monitor signs and symptoms of thrombotic events in patients at high risk of thrombosis [11]. Therefore, reversal by a specific antidote (as yet unavailable) is unlikely to change the priority given to conservative therapy, which was used successfully in this case.

The present case is the first reported case of DAH associated with edoxaban. DOACs, including edoxaban, should be prescribed with caution, especially for patients in a critical condition, as there is still no safe or efficacious reversal agent to stop factor Xa inhibitor-associated acute major bleeding.
